# Bidirectional regulation mechanism of TRPM2 channel: role in oxidative stress, inflammation and ischemia-reperfusion injury

**DOI:** 10.3389/fimmu.2024.1391355

**Published:** 2024-06-28

**Authors:** Peng Huang, Chaoyi Qu, Zhijian Rao, Dongzhe Wu, Jiexiu Zhao

**Affiliations:** ^1^ School of Kinesiology, Shanghai University of Sport, Shanghai, China; ^2^ Exercise Biological Center, China Institute of Sport Science, Beijing, China; ^3^ Physical Education College, Hebei Normal University, Shijiazhuang, China; ^4^ College of Physical Education, Shanghai Normal University, Shanghai, China; ^5^ Department of Exercise Physiology, Beijing Sport University, Beijing, China

**Keywords:** TRPM2 channels, calcium signal, oxidative stress, inflammation, ischemia-reperfusion

## Abstract

Transient receptor potential melastatin 2 (TRPM2) is a non-selective cation channel that exhibits Ca^2+^ permeability. The TRPM2 channel is expressed in various tissues and cells and can be activated by multiple factors, including endogenous ligands, Ca^2+^, reactive oxygen species (ROS) and temperature. This article reviews the multiple roles of the TRPM2 channel in physiological and pathological processes, particularly on oxidative stress, inflammation and ischemia–reperfusion (I/R) injury. In oxidative stress, the excessive influx of Ca^2+^ caused by the activation of the TRPM2 channel may exacerbate cellular damage. However, under specific conditions, activating the TRPM2 channel can have a protective effect on cells. In inflammation, the activation of the TRPM2 channel may not only promote inflammatory response but also inhibit inflammation by regulating ROS production and bactericidal ability of macrophages and neutrophils. In I/R, the activation of the TRPM2 channel may worsen I/R injury to various organs, including the brain, heart, kidney and liver. However, activating the TRPM2 channel may protect the myocardium from I/R injury by regulating calcium influx and phosphorylating proline-rich tyrosine kinase 2 (Pyk2). A thorough investigation of the bidirectional role and regulatory mechanism of the TRPM2 channel in these physiological and pathological processes will aid in identifying new targets and strategies for treatment of related diseases.

## 1 Introduction

Transient receptor potential (TRP) channels are a class of ion channels that contain TRP protein homologous sequences (1). They are widely distributed throughout the animal kingdom. In mammals, approximately 28 types of TRP channels have been identified, the majority of which are non-selective cation channels, with the exception of TRPV5 and TRPV6 (2, 3). Most TRP family ion channels are permeable to Ca^2+^ and can be activated by physical and chemical factors (4, 5). Some channels can also act as temperature receptors, which help the body sense changes in ambient temperature (6, 7), or as oxidative stress receptors, which mediate the body’s physiological activities (8–10). This multi-modal activation mechanism, which can be triggered by chemical, physical, and biological stimuli, enables the TRP family to play crucial roles in various physiological and pathological processes (4, 5). Transient receptor potential channel melastatin 2 (TRPM2) is a non-selective cation channel that has Ca^2+^ permeability. It was first discovered in 1998 and officially named as such in 2002 (11). The TRPM2 channel is widely expressed endogenously in tissues and cells, including the brain, heart, liver, skeletal muscle, lung, stomach, intestine, kidney, and pancreas (12–14). It is abundant in the brain, particularly in the hippocampus, substantia nigra (15, 16), hypothalamus (6, 7), striatum, and cerebral cortex, and expressed in various cell types, including microglia (17), astrocytes, neurons (18), endothelial cells, immune cells (19, 20), and cardiomyocytes (3, 15, 16, 21, 22).

Research has shown a strong correlation between the TRPM2 channel and oxidative stress, inflammation and ischemia–reperfusion (I/R). Activating the channel could increase intracellular Ca^2+^ levels, exacerbate oxidative stress and lead to cell death (23, 24). In certain circumstances, the entry of Ca^2+^ through TRPM2 channels can have a protective effect on tissues. In inflammation and I/R injury, the TRPM2 channel exhibits dual effects, that is, it can exacerbate tissue damage and protects tissues (25–29). However, the multifaceted role of TRPM2 in physiological and pathological processes have not been elucidated yet. This article provides a comprehensive review of the structure, regulatory mechanisms and diverse roles of the TRPM2 channel in physiological and pathological processes such as oxidative stress, inflammation and I/R. This work aims to gain a deep understanding of the physiological and pathological characteristics of the TRPM2 channel and offer new targets and strategies for treatment of relevant diseases.

## 2 Structure and biological characteristics of TRPM2 channel

**Figure 1:**
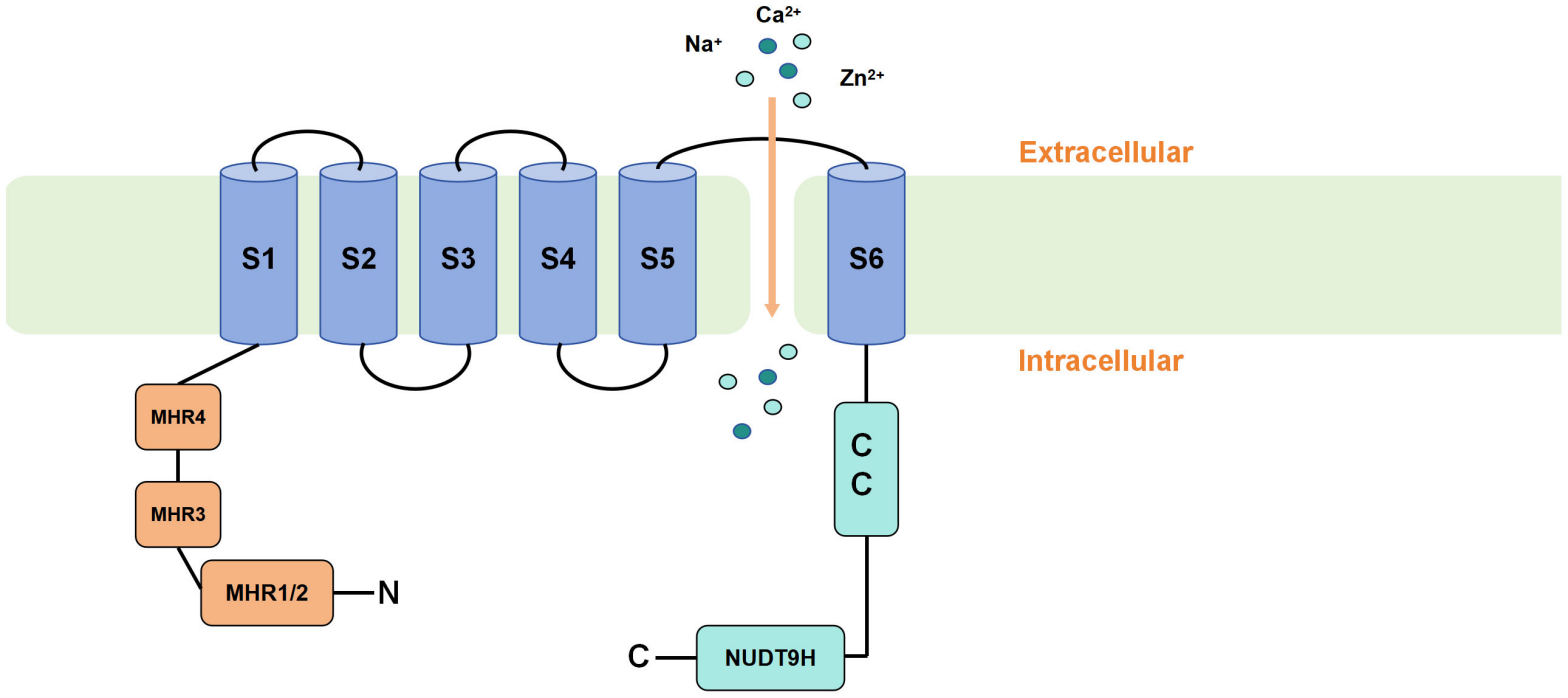
Schematic representation of TRPM2 membrane topology. TRPM2 consists of six transmembrane segments (TMS) (S1-S6) and intracellular N- and C-termini. The N-terminus contains TRPM homology regions (MHR1-4), while the C-terminus includes a coiled-coil tetramerization domain (CCR) and a unique NUDT9-H domain. Upon binding of ADPR to the MHR1/2 and NUDT9-H domains, TRPM2 is activated, causing the pore-forming loop between the S5 and S6 TMS to open and allow the influx of cations into the cell.

The human TRPM2 gene is located on chromosome 21q22.3 and consists of 32 exons. Its gene size is approximately 90 kB, and the molecular weight of mammalian TRPM2 is 170 kd (30). The TRP family has a similar overall structure. TRPM2 consists of six transmembrane segments (TMS) (S1–S6) and intracellular N- and C-termini, The N-terminus contains TRPM homology regions (MHR1-4) (31), while the C-terminus includes a coiled-coil tetramerization domain (CCR) and a NUDT9-H domain (32, 33). A pore-forming loop between the S5 and S6 TMS allows for the permeability of various ions. Upon activation of the TRPM2 channel, cations such as Zn^2+^, K^+^, Ca^2+^ and Na^+^ can be transported across the membrane through this channel (3). TRPM2 differs from other members of the TRP family because of the presence of a unique NUDT9-H domain in its C-terminus, which is similar to that of the mitochondrial NUDT9 enzyme. Additionally, cryo-electron microscopy (cryo-EM) studies have reported a TRPM homology region 1/2 (MHR1/2 domain) located at the N-terminus of the channel. Research has demonstrated that this domain also serves as a binding site for adenosine diphosphate ribose (ADPR). These two domains render TRPM2 sensitive to ADPR, and upon ADPR binding, the channel opens and thus allows cations to enter the cell (34–37) (**Figure 1**). In addition to the full-length TRPM2 (TRPM2-L), several different physiological splice variants have been identified, including TRPM2-S, TRPM2-AS, TRPM2-ΔN, TRPM2-ΔC, TRPM2-SSF and TRPM2-TE. These variants exhibit varying levels of activity and may potentially co-regulate the functional activity of the full-length TRPM2. However, the current understanding of the physiological functions and interactions among these splice variants is limited (38, 39).

## 3 TRPM2 regulation

The TRPM2 channel can be activated by endogenous ligands, Ca^2+^ and cluster of reactive oxygen species (ROS) (40, 41). These factors not only have the capability to independently activate the TRPM2 channel but also act synergistically. Endogenous activators of TRPM2 include ADPR, cyclic adenosine diphosphate ribose (cADPR), nicotinamide adenine dinucleotide (NAD^+^) and their metabolic derivatives (42, 43). ADPR is considered the most effective endogenous activator of TRPM2 due to its unique NUDT9-H domain (44). In mitochondria, NUDT9-ADPRase can convert NAD^+^ into ADPR (42). Besides mitochondrial sources, ADPR can also be generated in the nucleus through the poly (ADP–ribose) polymerase (PARP)/poly (ADP–ribose) glycohydrolase) (PARP/PARG) pathway when ROS accumulation leads to DNA damage, catalyzing the conversion of NAD^+^ to ADPR (45–47). The activation of TRPM2 is highly dependent on the levels of intracellular and extracellular Ca^2+^. In the absence of Ca^2+^, ADPR loses its activating effect on TRPM2 (48). This phenomenon may be due to an increased sensitivity of the TRPM2 channel to ADPR when Ca^2+^ is present (48). Recent cryo-EM studies have identified Ca^2+^ binding sites within the second and third TMS (S2-S3) of human TRPM2 (49), with similar highly conserved Ca^2+^ binding sites also found in other species including humans, zebrafish and Nematostella vectensis (49–51). Additionally, phosphatidylinositol 4,5-bisphosphate (PIP2) may also be involved in TRPM2 activation in the presence of ADPR and Ca^2+^. Research has shown that PIP2 plays a crucial role in the activation of TRPM2 orthologues in humans, zebrafish, and sea anemones (51–53). In addition to facilitating the activating effect of ADPR, Ca^2+^ can independently activate TRPM2 in the absence of ADPR (albeit with lower efficiency compared with ADPR) (54). Furthermore, Ca^2+^ is the only known agonist for certain TRPM2 splice variants (54), thereby emphasizing the importance of Ca^2+^ in TRPM2 activation. Apart from ADPR and NAD^+^, another cyclic metabolite of NAD^+^, cADPR, has also garnered significant attention. However, considerable debate exists regarding whether cADPR can bind to and gate TRPM2 channels, due to differences in experimental conditions and methodologies among different laboratories, leading to disparate results (43, 55–57). In addition to the aforementioned common endogenous agonists of TRPM2, recent studies have identified bilirubin as a new endogenous agonist of TRPM2, capable of directly binding and activating TRPM2 channels via a specific binding cavity in the transmembrane domain (58). Furthermore, certain analogs of ADPR have been identified as endogenous agonists of TRPM2. These encompass ADP-ribose-2’-phosphate (ADPRP) and 2’-Deoxyadenosine 5’-diphosphoribose (2dADPR), both demonstrating enhanced TRPM2 activation efficiency and evoking larger whole-cell currents relative to ADPR (59, 60). This discovery suggests a potentially fruitful avenue for future investigations into TRPM2 agonists.

The TRPM2 channel is activated by ROS and serves as a crucial oxidative stress sensor in the body (8–10). Although ROS are generally considered harmful, mitochondria release physiological levels of ROS under normal oxygen supply. These ROS function as signaling molecules, regulate numerous protein functions and play a vital role in maintaining normal physiological functions in the organism (61). Hydrogen peroxide (H_2_O_2_) is a crucial molecule involved in intracellular redox signaling. Micromolar levels of H_2_O_2_ as well as agents capable of generating ROS can activate TRPM2. However, controversy persists regarding whether H_2_O_2_ directly activates TRPM2 or operates through the PARP/PARG pathway in the nucleus or mitochondria to generate ADPR, which indirectly activates TRPM2. In addition, research has shown that ROS can induce the activation of protein kinase C and the phosphorylation of the Ser39 site on TRPM2-S. This event leads to the uncoupling of TRPM2-S from TRPM2, thereby relieving the inhibitory effect of TRPM2-S on TRPM2 and opening the TRPM2 channel (62).

TRPM2 channels act as thermosensors, regulating body temperature (6, 7). As early as 2008, studies observed that room temperature could influence H_2_O_2_-mediated opening of the TRPM2 channel (63). Temperatures exceeding 35°C can activate TRPM2 channel opening or facilitate its activation by ADPR and cADPR in rat insulinoma cells (41). TRPM2 serves as a thermosensor in the neural circuitry of the preoptic area involved in body temperature regulation and is activated when the body temperature exceeds 37°C (6). The activation and inhibition of TRPM2-positive neurons can respectively lead to a decrease and increase in body temperature, and downstream effects are associated with the release of oxytocin (64). TRPM2 channels in the brain respond to prolonged heat stimulation. The expression of TRPM2 mRNA in the embryonic mouse brain gradually increases under prolonged heat stimulation and affects embryonic development and neurogenesis (65). However, specific mechanisms governing the temperature activation of TRPM2 channels remain incompletely understood. The capacity of TRPM2 channels to respond to temperature renders them as sensors, which contribute to an organism’s perception of environmental temperature and regulation of body temperature processes.

Certain metal cations, such as Zn^2+^ and Cu^2+^, exert inhibitory effects on TRPM2 activation and serve as extracellular antagonists (66). Various drugs, including 12-deacetylscalaradial (DSD), clotrimazole (CTZ), anthranilic acid (ACA), flufenamic acid (FFA) and 2-aminoethoxydiphenyl borate (2-APB), can inhibit TRPM2 channel activity (67). However, these antagonists lack specificity for TRPM2. The activity of the TRPM2 channel is negatively regulated by cellular acidification (68). Exposure of intracellular and extracellular environments to acidic conditions (pH of 5 to 6) suppresses the activity of TRPM2 (69).

## 4 Bidirectional regulation of TRPM2 in oxidative stress

Oxidative stress refers to the disruption of the balance between oxidation and antioxidation in the body and leads to excessive generation or reduced scavenging of ROS or reactive nitrogen species and even cell and tissue damage when severe (70). Under normal physiological conditions, ROS are generated naturally by the mitochondrial electron transport chain during respiration. ROS act as signaling molecules and regulate various physiological functions in the human body, such as promoting cell survival, proliferation and differentiation (71). Under pathological conditions, ROS are produced by neutrophils and phagocytes involved in inflammation and infection. The decrease in the activity of the mitochondrial electron transport chain is induced by various factors and can contribute to increased ROS production (72). Continuous exposure to high levels of ROS damages nuclear DNA. Due to the involvement of the PARP/PARG pathway in DNA damage repair in the nucleus, this process is accompanied by the production of ADPR. Studies have demonstrated that the sensitivity of TRPM2 channels to activation under pathological levels of ROS is significantly increased, which is directly related to the activation of PARP/PARG (39, 73, 74).

### 4.1 TRPM2-mediated cell death induced by oxidative stress

The TRPM2 channel serves as a crucial oxidative stress sensor in the body and plays a significant role in various physiological and pathological processes (8–10). Upon activating TRPM2, oxidative stress leads to a sustained increase in cytoplasmic Ca^2+^ concentration, triggers inflammation and exacerbate cellular damage, ultimately resulting in cell death (23, 24). For example, the excessive use of acetaminophen may lead to hepatocyte death and may be associated with the pronounced increase in reactive ROS induced by acetaminophen and the substantial influx of Ca^2+^ into cells through TRPM2 (14, 75, 76). Acetaminophen induces a rapid increase in intracellular Ca^2+^ concentration and cation current in cultured rat and mouse hepatocytes. This response is inhibited by treatment with CTZ, ACA, and TRPM2-specific small interfering RNA (TRPM2-siRNA) (14). Consistent with *in vitro* experiments, injection of acetaminophen into wild-type (WT) mice leads to extensive liver necrosis and lymphocytic infiltration, accompanied by increased concentrations of hepatic enzymes alanine transaminase and aspartate transaminase in the blood. However, these damages are significantly reduced in TRPM2 knockout (KO) mice (14). This study provides evidence supporting that acetaminophen induces acute liver injury through the generation of ROS, which increases PARP-mediated ADPR production, subsequently activating TRPM2 and inducing Ca^2+^ signaling. The massive influx of Ca^2+^ into the cell upon TRPM2 activation mediates the acute liver damage caused by acetaminophen through downstream pathways. Further research has elucidated the molecular and signaling mechanisms by which TRPM2 mediates acetaminophen-induced cell death. It is proposed that the entry of Ca^2+^ through TRPM2 activates the calcium/calmodulin-dependent protein kinase II (CaMKII), which subsequently phosphorylates Beclin-1. This phosphorylation reduces the interaction between Beclin-1 and PIK3C3, thereby inhibiting autophagy while promoting apoptosis. Consequently, this enhances hepatocyte sensitivity to cell death (76). Yang et al. (77) revealed that TRPM2 expressed on the membrane of myocardial cells is involved in ROS-mediated myocardial cell death. Excessive ROS can induce the production of ADPR by increasing PARP levels and subsequently activate TRPM2. This activation leads to the excessive uptake of Ca^2+^ and Na^+^ by the mitochondria, causing mitochondrial membrane dysfunction, release of cytochrome C and activation of caspase-3. Endothelial cells covering the vascular wall are particularly susceptible to oxidative stress. *In vitro* experiments have shown that exposure to H_2_O_2_ or tumor necrosis factor-alpha (TNF-α-induced) ROS generation leads to the activation of TRPM2 channels in endothelial cells, subsequently inducing caspase-dependent apoptosis (increased activity of caspase3, caspase8, and caspase9), thereby reducing endothelial cell survival rates. However, these effects are mitigated or abolished following intervention with TRPM2 inhibitors or TRPM2-siRNA (19, 78, 79). These findings support the pivotal role of TRPM2 activation and TRPM2-mediated Ca^2+^ signaling in oxidative stress-induced endothelial cell death and endothelial barrier dysfunction, potentially implicating certain vascular diseases. Neurons in the brain are highly susceptible to oxidative stress damage, and ROS-induced neuronal death is closely associated with cognitive impairment and the pathogenesis of various brain diseases. TRPM2 is widely and abundantly expressed in the brain, and previous studies have found that TRPM2 mediates the process of ROS-induced neuronal death (80–82). Fonfria et al. (80) demonstrated the involvement of the TRPM2 channel in H_2_O_2_-induced neuronal death using rat striatal neurons. They found that the induction of TRPM2-siRNA or transient transfection of striatal neurons with a plasmid expressing TRPM2-S resulted in a reduction of H_2_O_2_-induced cell death. Additionally, treatment with the PARP inhibitor SB-750139 attenuated this cell death (80). These findings suggest that PARP-dependent activation of the TRPM2 channel mediates the process of H_2_O_2_-induced neuronal death. In cortical neurons of rats treated with H_2_O_2_, similar results were observed, and the study further revealed the crucial role of TRPM2-mediated Ca^2+^ influx in H_2_O_2_-induced neuronal death (82). Additionally, researchers utilized hippocampal neuron cultures from WT and TRPM2 KO mice and found that hippocampal neurons from WT mice exhibited concentration-dependent and duration-dependent cell death upon H_2_O_2_ intervention, whereas this cell death was significantly reduced in TRPM2 KO neurons. H_2_O_2_ also induces significant Zn^2+^ influx, lysosomal Zn^2+^ release, and lysosomal dysfunction, leading to mitochondrial dysfunction, ROS accumulation, and ultimately cell death (81). These consistent findings collectively suggest that the TRPM2 channel and its mediated excessive Ca^2+^ influx play a critical role in H_2_O_2_-induced neuronal death.

### 4.2 Protective effects of TRPM2 channel in oxidative stress

Contrary to the previous research findings, recent studies have revealed that under certain conditions, the entry of Ca^2+^ through the TRPM2 channel plays a significant physiological role in protecting various tissues from oxidative stress damage. When subjected to intraperitoneal injection of endotoxin, TRPM2 KO mice exhibit significantly lower survival rates than WT mice. These mice underwent severe oxidative stress and inflammatory reactions in their lungs (26). Further *in vitro* experiments showed that this phenomenon is caused by the entry of Ca^2+^ through TRPM2, which depolarizes the phagocyte cytoplasmic membrane and reduces ROS generation mediated by nicotinamide adenine dinucleotide phosphate (NADPH) oxidase (26). In cone neurons subjected to exogenous H_2_O_2_ intervention, inhibiting TRPM2 exacerbates oxidative stress damage to cells; this finding provides evidence that TRPM2 can protect neurons from oxidative stress injury (25). Moreover, TRPM2 mutations (P1018L) were identified in the brain tissues of patients with Guamanian amyotrophic lateral sclerosis and Parkinsonian dementia. Unlike the non-inactivating TRPM2, the P1018L mutant deactivates upon ADPR-induced channel opening, thereby restricting Ca^2+^ entry (63). This observation suggests that sustained Ca^2+^ influx triggered by activated TRPM2 channels is essential to maintain normal neuronal function. In the acute kidney injury model induced by chemotherapeutic drug cisplatin, the knockout of the TRPM2 gene exacerbates renal dysfunction and tissue damage. In TRPM2 KO mice and primary renal cells, the generation of mitochondrial cytochrome C and ROS increased, leading to intensified oxidative stress damage. However, the use of mitochondrial ROS scavenger Mito-tembo alleviates this damage. Hence, TRPM2 may mediate autophagy through a Ca^2+^/AKT/mTOR-dependent mechanism to maintain mitochondrial dynamics and protect the kidneys from oxidative stress injury (83). In contrast to the previous studies on TRPM2-mediated endothelial dysfunction induced by oxidative stress, recent research has revealed that TRPM2 exerts vasodilatory effects upon activation by H_2_O_2_. Experimental approaches involved the use of hypertensive mouse models with either WT or NOX4-knockout (NOX4 KO) genotypes, as well as a rat aortic endothelial cell model. Mechanistic pathways were validated through inhibition of PARP, NOX4, and TRPM2, as well as TRPM2-siRNA. It was demonstrated that NOX4-induced H_2_O_2_ production, via the PARP/PARG pathway, generated ADPR, which subsequently activated TRPM2-mediated Ca^2+^ influx, leading to activation of endothelial nitric oxide synthase (eNOS) and nitric oxide (NO) release, thereby exerting vasodilatory effects. These experiments elucidated the molecular mechanism underlying the blood pressure-lowering and vascular protective effects induced by NOX4 activation, while also suggesting that TRPM2 may hold significant implications in ameliorating diseases associated with endothelial dysfunction, such as hypertension (84).

**Figure 2:**
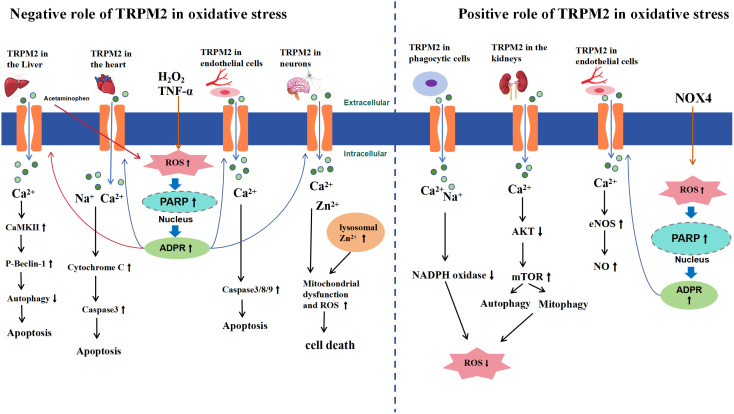
Schematic Representation of TRPM2-Mediated Signaling Pathways in Oxidative Stress. The left side of the diagram illustrates the negative effects of TRPM2 activation under oxidative stress, while the right side highlights the positive effects. In the liver, heart, endothelial cells, and brain neurons, extracellular H_2_O_2_ or TNF-α leads to intracellular ROS accumulation (acetaminophen-induced ROS accumulation is specific to liver conditions), inducing activation of PARP and PARG enzymes in the nucleus, which convert NAD^+^ to ADPR, thereby activating TRPM2. Subsequently, a large influx of Ca^2+^, Zn^2+^, and Na^+^ occurs, which, depending on the tissue, triggers apoptosis or cell death through activation of signaling pathways such as CaMKII and caspase (left side). In the kidney and phagocytic cells, TRPM2 activation-mediated Ca^2+^ and Na^+^ influx promotes autophagy through the AKT-mTOR signaling pathway or inhibits NADPH to reduce ROS production. In endothelial cells, NOX4 generates ADPR via the ROS/PARP pathway to activate TRPM2, which can activate the eNOS/NO signaling pathway, leading to vasodilation (right side).

In summary, under physiological and pathological conditions, the activation of TRPM2 may exacerbate or alleviate oxidative stress damage (**Figure 2**), which may depend on the following factors: from a cellular and tissue-specific perspective, TRPM2 activation exacerbates oxidative stress-induced hepatocyte and cardiomyocyte damage, with mechanistic similarities involving ROS/PARP pathway-mediated ADPR production leading to TRPM2 activation, subsequent massive Ca^2+^ influx, and downstream effects resulting in cell apoptosis; In mouse renal tissue and phagocytic cells, TRPM2 activation appears to reduce ROS production. However, it is important to acknowledge the limitations of this conclusion. Firstly, it is uncertain whether this effect is specific to oxidative stress induced by endotoxin and cisplatin. Secondly, there is limited research on the role of TRPM2 in mediating oxidative stress-induced damage in renal tissue and phagocytic cells, necessitating further investigation to elucidate the relationship between TRPM2 and oxidative stress in these contexts; In neuronal cells, there is controversy surrounding the research findings, but the majority support that TRPM2 activation exacerbates cellular oxidative stress damage. However, differences between experimental results may be attributed to variations in extracellular environments and the use of nonspecific inhibitors. In endothelial cells, ROS-triggered TRPM2 activation may manifest dual effects, either promoting endothelial cell apoptosis or inducing vasodilation through NO release. However, whether both effects are simultaneously activated remains unclear, further highlighting the complexity of TRPM2 downstream pathways. Additionally, variations in the genetic background of mice used in different experiments may lead to disparate outcomes within the same tissue or cell types. Lastly, the lack of specific inhibitors for TRPM2 could potentially introduce bias into experimental results. Therefore, further comprehensive research is warranted to assess the specific role of TRPM2 in oxidative stress.

### 4.3 TRPM2 in diseases associated with oxidative stress

The Ca^2+^ influx induced by ROS activation of the TRPM2 channel significantly alters intracellular Ca^2+^ concentration, thereby affecting downstream effects including cell signaling, proliferation, apoptosis, and even cell death. TRPM2 occupies a pivotal position in the ROS and Ca^2+^ systems, and its activation-mediated cell death effects may link oxidative stress-induced pathological factors to related diseases. Research indicates that dysfunctional or dysregulated TRPM2 function has been associated with a range of pathological conditions, including neurodegenerative diseases and diabetes. Oxidative stress is associated with neurodegenerative diseases and a characteristic feature of physiological aging in the brain (85–87). Glutathione (GSH) is a crucial endogenous antioxidant in the body, and a decrease in its levels results in reduced cellular antioxidant defense and oxidative stress (88). Intraneuronal GSH levels decrease during aging, which may be linked to the expression of TRPM2. *In vitro* experiments demonstrated that inhibiting GSH synthesis results in reduced expression and significantly increases TRPM2 activity. TRPM2 may have a significant role in age-related neurodegenerative diseases (89). Alzheimer’s disease (AD) is a common age-related neurodegenerative disease characterized by progressive cognitive decline (90), deposition of brain neuronal β-amyloid (Aβ) plaques, increased reactive ROS, and intracellular Ca^2+^ overload (91). Oxidative stress alters Ca^2+^ homeostasis in AD patients and animal models (92, 93), leading to an increased concentration of intracellular free Ca^2+^, which subsequently induces mitochondrial membrane depolarization and cell death (94). In AD mouse models, TRPM2 KO significantly ameliorates age-related memory impairments, synaptic loss, and activation of microglial cells (18). *In vitro* experiments also demonstrate that prolonged exposure of hippocampal neuronal cells to pathological concentrations of Aβ42 results in lysosomal Zn^2+^ release, mitochondrial Zn^2+^ accumulation, mitochondrial fragmentation, and substantial production of mitochondrial ROS, accompanied by extensive neuronal cell death. However, these aberrant changes induced by Aβ42 are abolished following intervention with 2-APB or TRPM2 KO. These experimental findings suggest a crucial role of TRPM2 in mediating Aβ-induced AD pathology (95). Furthermore, it is hypothesized that Aβ42 activates protein kinase C (PKC) and NADPH oxidase (NOX) to generate ROS, inducing ADPR production via the PARP/PARG pathway, thereby activating the TRPM2 channel. Subsequently, this leads to lysosomal dysfunction and increased lysosomal Zn^2+^ release, mitochondrial Zn^2+^ accumulation resulting in mitochondrial fragmentation, dysfunction, release of cytochrome C and mitochondrial ROS, ultimately leading to cell death through the apoptotic (95). The substantial Ca^2+^ influx induced by TRPM2 activation also mediates Aβ-induced cerebrovascular dysfunction. This mechanism is likely triggered by Aβ-induced oxidative-nitrosative stress in cerebral endothelial cells, causing DNA damage and excessive ADPR production via the PARP/PARG pathway. The activation of TRPM2 on endothelial cells leads to Ca^2+^ overload, ultimately impairing cerebral blood flow (96). Studies have found that patients with Type I bipolar disorder exhibit elevated baseline intracellular Ca^2+^ levels, and a susceptibility locus on chromosome 21q22.3, which includes the TRPM2 gene region, has been identified, suggesting a potential link between TRPM2 and bipolar disorder (97, 98). Additionally, excessive activation of TRPM2 in neurons may also be associated with the development of long-term depression (LTD) and Parkinson’s disease (PD) (99, 100). In addition to neurons, TRPM2 has also been found to be expressed in pancreatic β-cells. Pancreatic β-cells have relatively weak antioxidant capabilities and are susceptible to oxidative stress damage, and a reduction in their number and dysfunction can lead to hypoinsulinemia, hyperglycemia, or diabetes (99). *In vitro* experiments using insulin-secreting cell lines (RIN-5F and INS-1 cells) have demonstrated that the activation of PARP-dependent TRPM2 channels mediates H_2_O_2_-induced apoptosis, accompanied by excessive Ca^2+^ release from intracellular lysosomes, ultimately leading to cell death. TRPM2 KO or the use of TRPM2 inhibitors can mitigate this cell death (101, 102). In streptozotocin (STZ)-induced rodent models of diabetes have confirmed this phenomenon, showing extensive loss of pancreatic β-cells and significantly elevated fasting blood glucose levels in mice, which are ameliorated in TRPM2 KO mice. PARP-dependent TRPM2 activation mediates Ca^2+^ influx and substantial lysosomal Zn^2+^ release, disrupting ionic homeostasis in pancreatic β-cells and triggering apoptosis through downstream effects (101). This indicates that TRPM2 may play a critical role in the pathogenesis of (type 1 diabetes) T1D and (type 2 diabetes) T2D by mediating oxidative stress-induced pancreatic β-cell death. Current research findings suggest that TRPM2 activation could be a significant factor in the onset and progression of neurodegenerative diseases or diabetes. Thus, TRPM2 may represent a promising new target for the treatment of certain ROS-related diseases.

### 4.4 Activation of TRPM2 promotes cancer cell survival

SH-SY5Y cells are a human neuroblastoma cell line initially derived from a neuroblastoma. They are commonly used *in vitro* to study various aspects of neuronal cell growth, differentiation, apoptosis, and mechanisms of neurodegenerative diseases. In SH-SY5Y cells, the activation of TRPM2 appears to play a critical role in promoting cell proliferation and survival. In SH-SY5Y cells subjected to low-dose H_2_O_2_ intervention, the expression of the splicing variant TRPM2-S, which can inhibit TRPM2 channel activity, results in decreased intracellular Ca^2+^ influx and elevated levels of ROS, leading to a reduction in cell survival rates (103). Chen et al. (103)study further elucidated that SH-SY5Y cells expressing TRPM2-S exhibited increased cell death upon exposure to H_2_O_2_, whereas cells expressing TRPM2-L were able to mitigate moderate oxidative stress-induced damage and reduce cell death by upregulating levels of forkhead box o3a (FOXO3a) and superoxide dismutase 2 (SOD2). Moreover, TRPM2 enhanced the growth capacity of SH-SY5Y cells. Besides its role in reducing cellular oxidative stress and enhancing antioxidant defenses to protect against cell death, TRPM2 was found to promote the growth and survival of SH-SY5Y cells through modulating autophagy (103). Autophagy is a lysosome-mediated degradation process and serves as a crucial mechanism for promoting cell survival (104). Additionally, autophagy plays a pivotal role in tumor growth by clearing damaged cellular components such as DNA and mitochondria, controlling ROS levels, enhancing cellular antioxidant capacity, and promoting tumor growth and survival (105–107). The absence of TRPM2 influences cellular autophagy (10, 83, 108, 109). Research that used CRISPR/Cas9 technology to knock out TRPM2 in SH-SY5Y neuroblastoma cells revealed inhibited cell growth. After doxorubicin intervention, oxygen consumption and ATP generation decrease, causing cellular bioenergetic impairment. Moreover, mitochondrial ROS production significantly increases. Pre-treatment with the antioxidant Mito-tembo reduces ROS levels and protects cellular viability (108). TRPM2 plays a crucial role in regulating the expression of hypoxia-inducible factor-1α/2α(HIF-1α/HIF-2α) and their downstream signaling pathways. The underlying mechanisms may involve factors, such as FOXO3a-SOD2, cytochrome c oxidase subunit 4.1/4.2 (COX4.1/4.2), BCL2/adenovirus E1B 19 kDa interacting protein 3 (BNIP3) and NADH:ubiquinone oxidoreductase subunit a4 like 2 (NDUFA4L2). SOD2 is an antioxidant enzyme located within the mitochondria. BNIP3 is a crucial factor in mitochondrial autophagy, and its reduction can lead to decreased mitophagy, resulting in the accumulation of dysfunctional mitochondria and an increase in ROS. The expression of NDUFA4L2 can reduce ROS production by affecting the activity of respiratory chain complex I. These downstream factors contribute to the reduction in intracellular ROS levels and protect cells from damages caused by oxidative stress (72). The above studies indicate that TRPM2 plays a crucial role in the proliferation and survival of SH-SY5Y cells by modulating intracellular ROS levels, antioxidant capacity, mitochondrial function, and autophagic capability.

In addition to neuroblastoma cells, recent studies have confirmed similar regulatory mechanisms of TRPM2 in acute myeloid leukemia cells and gastric cancer cells. Chen et al. (10)revealed that the knockout of TRPM2 in U937 cells suppressed cell proliferation and increased the sensitivity to atorvastatin intervention. In TRPM2 KO cells, mitochondrial membrane potential (ψm) and mitochondrial calcium uptake capacity significantly decreased. Mitochondrial function, including oxygen consumption rate and ATP production, were impaired, and the level of ROS significantly increased. Furthermore, the levels of nuclear factor erythroid 2-related factor 2 and transcription factors including HIF-1α/2α and FOXO3a decreased. Overall, the cellular antioxidant capacity was diminished. In TRPM2-deficient cells, there is a reduction in the key transcription factors ATF4 and cAMP response element-binding protein (CREB), which are essential for autophagosome biogenesis, and he levels of autophagy-related proteins such as unc-51-Like autophagy activating kinase 1, autophagy-related 7 and autophagy-related 5 decreased, leading to autophagy suppression. The restoration or enhancement of TRPM2 expression elevated cell viability as well as cellular proliferation and autophagic capability. Moreover, increased TRPM2 expression mitigated the increase in ROS level (10). Knockout of the TRPM2 gene in AGS and MKN-45 cells resulted in autophagy inhibition, impeded cell proliferation, promoted apoptosis and induced mitochondrial dysfunction characterized by reduced mitochondrial basal and maximal oxygen consumption rates as well as decreased ATP production. TRPM2, through the c-Jun N-terminal kinase (JNK) signaling pathway, modulates autophagy and the expression of autophagy-related proteins, including autophagy-related genes, microtubule-associated protein 1A/1B-light chain 3A/BII (LC3A/BII) and BNIP3. This regulation maintains mitochondrial energy metabolism, reduces cell sensitivity to doxorubicin and mitigates ROS generation (109). These data indicate that TRPM2 plays a crucial role in the proliferation and survival of the aforementioned cells by modulating key transcription factors and target genes involved in mitochondrial function, bioenergetics, antioxidant capacity and autophagy.

**Figure 3:**
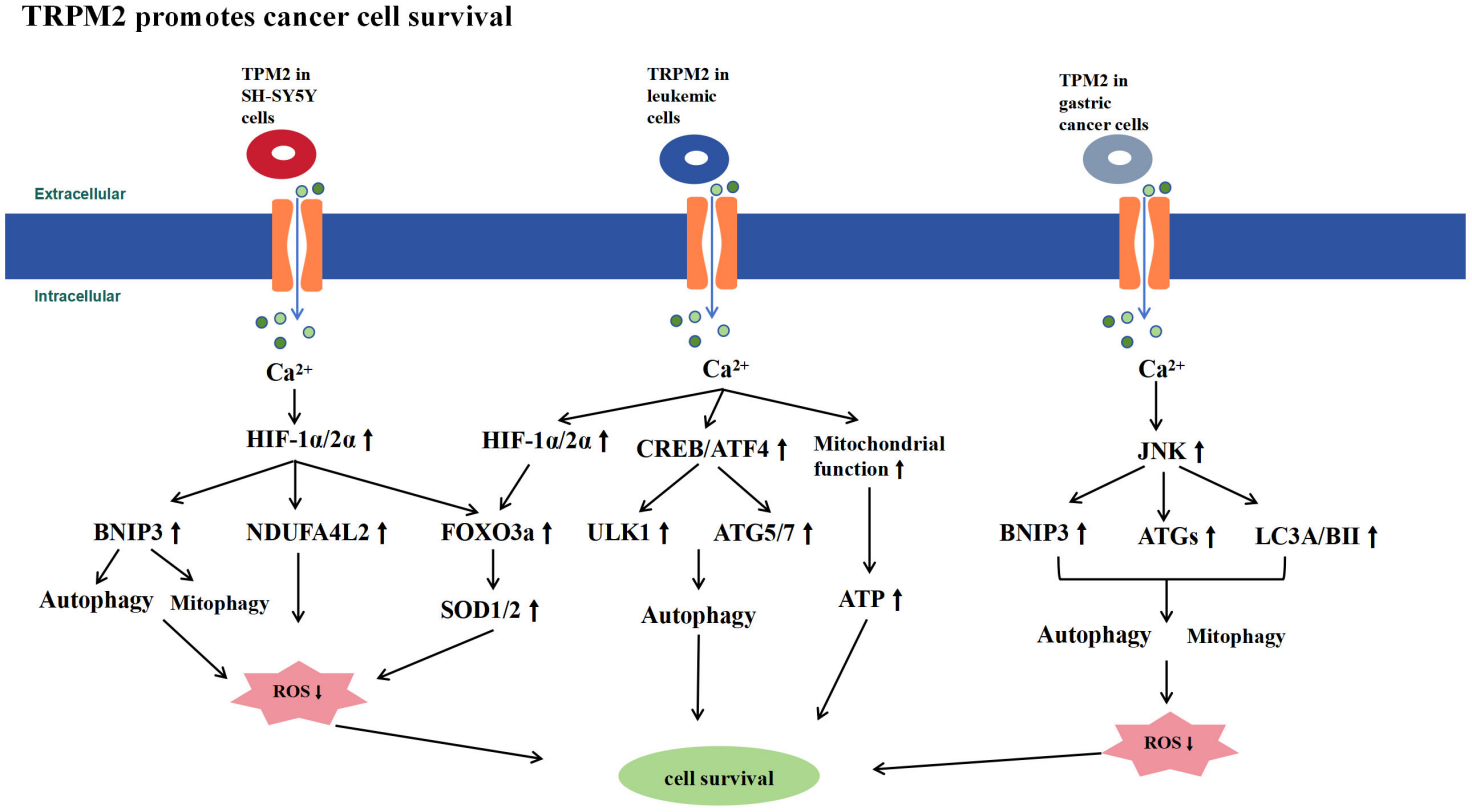
Schematic Representation of Signaling Pathways Promoting Tumor Cell Survival by TRPM2. TRPM2 is highly expressed in tumor cells and promotes their survival by controlling Ca^2+^ influx, which activates signaling pathways such as HIF-1α/2α, ATF4, CREB, and JNK. This activation enhances mitochondrial function, increases ATP production, promotes autophagy, reduces ROS levels, and boosts antioxidant capacity, thereby supporting tumor cell survival.

High TRPM2 expression in various cancers suggests that TRPM2 promotes tumor cell survival (**Figure 3**). Studies using multiple tumor cell models have shown that inhibiting TRPM2 increases cell death and sensitivity to doxorubicin. TRPM2 supports tumor cell survival by enhancing mitochondrial function, increasing ATP production, promoting autophagy, reducing ROS levels, and boosting antioxidant capacity. This mechanism ensures tumor cell survival under high ROS conditions while maintaining mitochondrial bioenergetics to meet energy demands. However, whether this effect is exclusive to tumor cells remains unclear and requires further experimental investigation. Although the activation of TRPM2 by ROS in tumor cells is not desirable due to its cell survival-promoting effects, it suggests that pharmacological inhibition of TRPM2 in cancer could be a novel and highly promising therapeutic approach.

## 5 Bidirectional regulation of TRPM2 in inflammation

ROS-induced Ca^2+^ influx plays a crucial role in the immune system (110–112), being associated with the activation of many inflammasomes and inflammatory factors, as well as the expression of their downstream signaling pathways (113–116). TRPM2 serves as a critical link between ROS and Ca^2+^ influx and is expressed in various inflammatory cells, including monocytes, neutrophils, and macrophages (117, 118). Therefore, TRPM2 may significantly influence inflammation by modulating immune cell functions.

### 5.1 Negative role of TRPM2 in inflammation

Ca^2+^ entry through the TRPM2 channel can regulate the production of ROS-induced monocyte chemotactic factors (113, 117). In human U937 monocytes, H_2_O_2_ causes ADPR-mediated opening of TRPM2 channels, leading to Ca^2+^ influx. This activates Pyk2 and Ras-GTPase, amplifying extracellular signal-regulated kinase (Erk) signaling and promoting nuclear translocation of nuclear factor-kappa B (NF-κB), which results in CXCL8 production. In a dextran sodium sulfate (DSS)-induced ulcerative colitis model, TRPM2 KO mice showed reduced CXCL2 expression, decreased neutrophil infiltration, and attenuated colonic inflammation (113). Thus, inhibiting TRPM2 channel function may represent a novel strategy for treating inflammatory diseases. Chen et al. (119)revealed that the genetic ablation of TRPM2 in microglial cells mitigated kainic acid-induced activation of microglia and generated inflammatory factors, which are common neuroinflammatory manifestations in epilepsy. This phenomenon was not observed in TRPM2-depleted astrocytes. Experimental evidence further demonstrated that the knockout of TRPM2 operates through the AMPK/mTOR pathway to modulate autophagy, thereby suppressing the activation of microglial cells. Chronic inflammation in the brain constitutes a mechanistic and characteristic feature in the pathogenesis of epilepsy and multiple sclerosis. In rodent models of pilocarpine-induced epilepsy and cuprizone-induced multiple sclerosis, the deletion of TRPM2 reduced cytokine levels and attenuated inflammasome engagement within murine brain tissue. This reduction was associated with diminished levels of activation in neuroglial cells, consequently ameliorating the severity of neuroinflammation (120). Additionally, the knockout of TRPM2 confers protection against hepatic cell damage induced by I/R and oxygen-glucose deprivation/reoxygenation (OGD/R). This protective effect is possibly likely associated with the activation of autophagy and the suppression of the NLR family pyrin domain containing 3 (NLRP3) inflammasome pathway (121). Analysis of ovarian cancer-associated transcriptomic and clinical data obtained from The Cancer Genome Atlas, Genotype-tissue Expression (GTEx) and Gene Expression Omnibus (GEO) databases reveal a robust positive correlation in the expression of TRPM2 with the NLRP3, NLR family CARD domain containing 4, nucleotide-binding oligomerization domain 1, nucleotide-binding oligomerization domain 2 interleukin 1 beta and gasdermin D. The increase in the expression of TRPM2 may be strongly associated with adverse prognosis in ovarian cancer, suggesting its potential utility as a novel immunotherapeutic target to enhance the overall survival of patients with ovarian cancer (122). The aforementioned studies suggest that the activation of the TRPM2 channel leads to sustained Ca^2+^ influx, induces inflammasome activation and produces inflammatory and chemotactic factors. This process emerges as a crucial factor in exacerbating inflammatory responses and tissue damage. The inhibition of TRPM2 channel functionality emerges as a potential target for ameliorating cellular inflammation.

### 5.2 Positive role of TRPM2 in inflammation

A substantial body of current research indicates that activating the TRPM2 channel inhibits inflammatory responses (23, 26, 29, 123–125). Research indicates that the survival rate of TRPM2 KO mice significantly decreases compared with WT mice following intraperitoneal injection of endotoxin (LPS). The deletion of TRPM2 enhances the expression levels of chemokine ligand 2, IL-6 and TNF-α in the lungs of mice, thereby exacerbating the inflammatory response. In the response of phagocytic cells to infection, the production of NADPH oxidase-dependent ROS plays a crucial role in inflammatory mechanisms (126, 127). Activation of TRPM2 may inhibit this ROS production, thereby mitigating LPS-induced pulmonary inflammatory damage (26). The knockout of TRPM2 enhances neutrophil-mediated vascular inflammatory responses. TRPM2-/- mice exhibited a significant increase in neutrophil accumulation at the site of intraperitoneal LPS injection. This study suggests that TRPM2 can sense ROS generated by neutrophils and inhibit neutrophil migration. In this process, the oxidation of the N-terminal Cys549 residue of TRPM2 induces its binding to formyl peptide receptor 1 (FPR1), thereby inhibiting FPR1 internalization and signaling. This inhibition impedes neutrophil migration and alleviates neutrophil-induced inflammatory damage (123).

TRPM2 can also suppress inflammatory response induced by bacterial infections. In a murine model of sepsis induced by cecal ligation and puncture (CLP), compared with WT mice, TRPM2 KO mice exhibited a significantly increase in mortality rate. This finding was characterized by elevated bacterial burden in the blood, lungs, liver and spleen. The mortality rate was associated with bacterial burden, organ injury and systemic inflammation. Overall, TRPM2 enhances the antimicrobial capability of immune cells to bolster the host’s anti-inflammatory capacity. Furthermore, TRPM2 potentially regulates the expression of heme oxygenase-1 by controlling Ca^2+^ influx, consequently promoting macrophage autophagy to enhance bacterial clearance (124, 125). In a murine sepsis model induced by intraperitoneal injection of Escherichia coli, TRPM2-/- mice exhibited a significantly increase in mortality rate and bacterial burden compared with WT mice, thereby exacerbating inflammatory responses. The absence of TRPM2 impaired the maturation of phagolysosomes, impeded their fusion with lysosomes in peritoneal macrophages and reduced the bactericidal capacity of macrophages. Increasing the intracellular Ca^2+^ concentration restored the bactericidal activity of macrophages, indicating that TRPM2 regulates the maturation of phagolysosomes in macrophages by controlling intracellular Ca^2+^ concentration and plays a crucial role in host defense against bacterial infections (128). Qian et al. (129) utilized TRPM2 KO neutrophils and TRPM2 KO mice in their study; they reported that TRPM2 induced the phosphorylation of p38 mitogen-activated protein kinase (MAPK) in neutrophils by mediating Ca^2+^ influx and regulated the release of elastase. This process enhances the bactericidal capacity of neutrophils. Research also revealed that TRPM2-/- mice are highly susceptible to infection with Listeria monocytogenes (Lm); post-infection decreased the levels of interleukin-12 and interferon-γ (23). In the same Lm model, TRPM2-/- mice presented with septic shock during infection and increased serum levels of TNF-α, IL-6 and IL-10. This study suggests that the activation of TRPM2 in neutrophils is beneficial in suppressing Lm dissemination and preventing neutrophil-mediated tissue damage, thereby contributing to the alleviation of local and systemic inflammatory levels (130). Furthermore, research has investigated the role of TRPM2 in macrophages by using Helicobacter pylori infection as a model of chronic inflammation. In comparison with WT mice, macrophages from TRPM2-/- mice exhibited an inability to regulate intracellular Ca^2+^ levels when stimulated with H. pylori. This phenomenon resulted in Ca^2+^ overload, wherein excessive intracellular Ca^2+^ enhanced the activities of MAPK and NADPH oxidase. The heightened activities of these components triggered macrophages to produce increased levels of ROS and inflammatory mediators, thereby exacerbating gastric inflammation in the mice. Hence, TRPM2 plays a regulatory and anti-inflammatory role in gastric inflammatory response (29).

**Figure 4:**
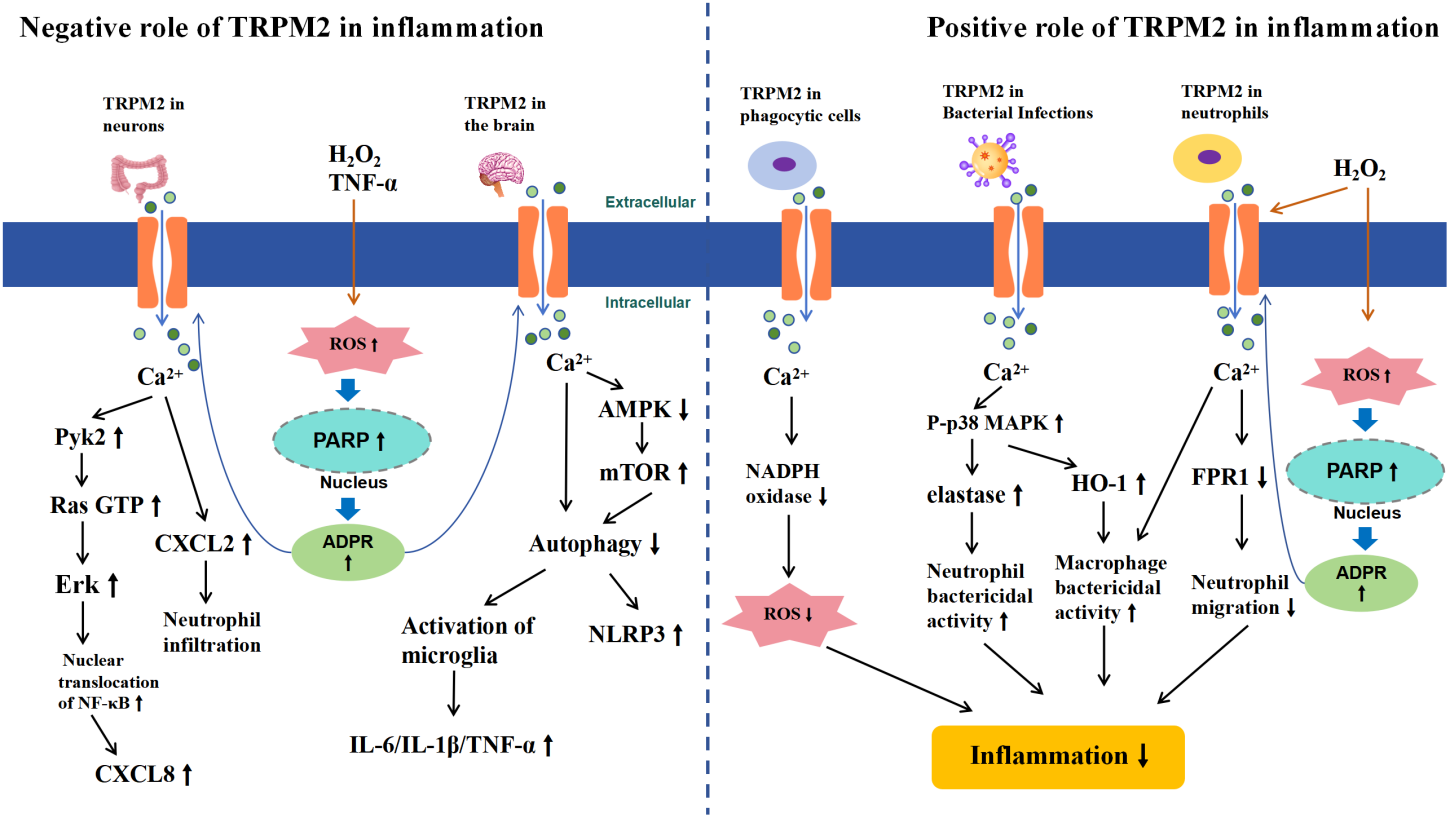
Schematic Representation of TRPM2-Mediated Signaling Pathways in Inflammation. The left half illustrates the negative role of TRPM2 activation in inflammation. In chronic inflammation of the gut and brain, extracellular H_2_O_2_ or TNF-α leads to intracellular ROS accumulation, inducing the activation of PARP and PARG enzymes in the nucleus, which convert NAD^+^ to ADPR, thereby activating TRPM2. Subsequently, Ca^2+^ influxes into the cells, triggering CXCL secretion in the gut through the activation of Pyk2-Erk signaling pathways and increasing inflammatory cytokines and inflammasomes in the brain by inhibiting the AMPK/mTOR signaling pathway, which suppresses autophagy (left half). The right half illustrates the positive role of TRPM2 activation in inflammation. In inflammatory responses caused by macrophages, neutrophils, or bacterial infections, TRPM2-mediated Ca^2+^ influx reduces ROS production by inhibiting NADPH, decreases neutrophil migration by inhibiting FPR1, or enhances the bacterial clearance ability of immune surveillance cells by activating the p38MAPK signaling pathway, thereby reducing inflammation (right half).

Thus, TRPM2 exhibits a dual role in the immune system, acting both pro-inflammatory and anti-inflammatory (**Figure 4**), with various factors contributing to this outcome. In intestinal inflammation such as ulcerative colitis and chronic brain inflammation like epilepsy, TRPM2 activation may exacerbate the inflammatory response and tissue damage by causing excessive Ca^2+^ influx, leading to increased production of inflammatory cytokines and inflammasomes, or by inducing glial cell activation. Conversely, in LPS-induced inflammation, TRPM2 activation can mitigate the inflammatory response by inhibiting NADPH oxidase-dependent ROS production and suppressing neutrophil aggregation and migration. Additionally, in the CLP model or inflammation caused by bacterial infections such as Lm, E. coli, and H. pylori, TRPM2 channel activation was found to suppress the inflammatory response, likely due to enhanced bacterial clearance by macrophages and neutrophils. This anti-inflammatory effect is not only effective in local tissues but also in reducing systemic inflammation levels. Therefore, the different roles of TRPM2 in inflammation are determined by the varying factors that trigger the inflammatory response, which may be related to the complex downstream signaling pathways of TRPM2. It remains unclear whether the anti-inflammatory effects of TRPM2 activation are limited to LPS and bacterial infection-induced inflammation. Future research is needed to further investigate the effects of TRPM2 activation in different inflammatory responses to fully understand the relationship between TRPM2 and inflammation.

## 6 Bidirectional regulation of TRPM2 in I/R

I/R is a complex process where tissue or organ blood flow is restricted and subsequently restored. Factors such as ischemia and hypoxia can lead to tissue damage and functional impairment. This phenomenon results in events, such as Ca^2+^ overload, oxidative stress and inflammatory responses, leading to cell death (131). I/R events may occur during myocardial infarction, stroke, acute kidney injury and organ transplantation. Oxidative stress and increased ROS production during cerebral I/R may activate non-selective cation channels, such as TRPM2, contributing to neuronal damage post I/R (131–133). ROS and intracellular Ca^2+^ overload are significant contributors to myocardial I/R injury, implicating the potential pivotal role of TRPM2 channels in these physiological and pathological processes.

### 6.1 Negative role of TRPM2 in I/R

In the heart, physiologically released ROS from the mitochondria may activate TRPM2 channels and provide sustained Ca^2+^ influx that is essential for normal cellular physiological functions (134). However, under pathological conditions such as I/R, an extensive influx of Ca^2+^ can lead to Ca^2+^ overload. The concomitant surge in ROS triggers oxidative stress, resulting in the opening of the mitochondrial permeability transition pore and caspase cascade activation, leading to cell death (77). TNF-α is a crucial pro-inflammatory factor involved in I/R injury and exacerbates cardiomyocyte death (135). Roberge et al. (136) reported that TNF-α induces a nonspecific cation current in isolated adult mouse ventricular myocytes, which can be inhibited by TRPM2 inhibitors, namely, CTZ and FFA. TNF-α activation of PARP-1 and TRPM2 results in a significant influx of intracellular Ca^2+^, potentially leading to mitochondrial Ca^2+^ overload, an increase in mitochondrial ROS, activation of caspase-8 and ultimately cardiomyocyte cell death (136). Conversely, inhibiting TRPM2 expression markedly reduces TNF-α-induced intracellular Ca^2+^ elevation and attenuates cardiomyocyte cell death. *In vivo* experiments revealed that, compared to WT mice, TRPM2-KO mice exhibited a significantly reduced myocardial infarct size and improved myocardial contractile function after left anterior descending coronary artery ligation and reperfusion. Additionally, the number of neutrophils and the activity of myeloperoxidase (MPO) in the I/R region were decreased. Neutrophils are a major source of pro-inflammatory mediators such as TNF-α and ROS, which exacerbate I/R-induced myocardial injury (137). These *in vivo* and *in vitro* findings indicate that TRPM2 plays a crucial role in mediating myocardial injury induced by I/R.

Depending on the severity and duration of ischemia, cerebral I/R can lead to varying degrees of neuronal dysfunction and death. Neuronal death during reperfusion, known as delayed neuronal death, is a major cause of ischemic brain injury (138). Studies have shown that TRPM2 activation plays a critical role in delayed neuronal death induced by I/R. During OGD/R, interventions with TRPM2 inhibitors ACA, CTZ, FFA, and 2-APB, as well as TRPM2-shRNA, significantly reduce cortical and hippocampal neuronal death and enhance cell viability (139–141). Studies have also demonstrated that genetic deletion of TRPM2 can alleviate hypoxic-ischemic brain injury in neonatal mice, characterized by reduced post-I/R cerebral infarct size and improved sensorimotor deficits (142). Of note, the efficacy of TRPM2 inhibition in ameliorating I/R brain injury appears to exhibit pronounced sex differences. Treatment with TRPM2 inhibitors, TRPM2-shRNA, or genetic ablation of TRPM2 expression did not significantly diminish delayed neuronal death in female mice following cardiac arrest-resuscitation (CA-R), reduce cerebral infarction in female mice subjected to middle cerebral artery occlusion-reperfusion (MCAO-R), or decrease delayed neuronal death in female mouse cortical and hippocampal neurons induced by OGD/R *in vitro (* 139–141). The mechanistic basis for these gender-dependent disparities in treatment efficacy remains unclear, possibly related to differential hormone levels, higher male sensitivity to ADPR (143), or activation of distinct downstream signaling pathways following I/R.

Ischemic stroke manifests as focal cerebral ischemia, characterized by excessive release of the neurotransmitter glutamate, leading to overactivation of NMDA receptors and subsequent neuronal intracellular Ca^2+^ overload along with excessive ROS production (144). Studies have demonstrated the pivotal role of NMDA receptor expression and downstream signaling in TRPM2-mediated cerebral ischemic stroke (144, 145). In the hippocampal neurons of TRPM2-deficient mice, the expression of the N-methyl-D-aspartate receptor (NMDAR) subunit GluN2A is significantly upregulated, while the expression of GluN2B decreases. The activation of TRPM2 has dual effects on neuronal survival following brain I/R. On the one hand, TRPM2 activation inhibits GluN2A activity, leading to a reduction in Ca^2+^ influx through GluN2A and suppressing downstream pro-survival signaling pathways, including the PI3K-Akt pathway and MAPKK-ERK1/2 pathway. This inhibition results in an increased expression of the pro-apoptotic factor GSK-3β downstream of protein kinase B (Akt). On the other hand, TRPM2 activation promotes an increase in the postsynaptic density protein 95 expression, consequently activating the GluN2B subunit. This activation, in turn, induces cell death by inhibiting the phosphorylation of ERK1/2. The combined action of these mechanisms mediated by TRPM2 contributes to neuronal death following brain I/R (146, 147). Recent studies have found that TRPM2 can directly interact with GluN2A, selectively increasing the activity and the surface expression of NMDAR subunits by recruiting PKCγ (148). TRPM2 enhances NMDAR-induced excitotoxicity through its physical and functional coupling with extrasynaptic NMDARs. Interventions targeting the uncoupling of TRPM2-NMDAR can effectively mitigate ischemic stroke damage (149). Additionally, uncoupling TRPM2 from PKCγ has been shown to reduce excitotoxicity and neuronal death in both *in vitro* and *in vivo* experiments. Thus, the TRPM2-PKCγ uncoupling may also represent an effective therapeutic strategy for reducing NMDAR-mediated excitotoxicity in ischemic stroke (150). Research has found that TRPM2 in brain endothelial cells exacerbates neuronal death during ischemic stroke by promoting Ca^2+^ influx and interacting with CD36, thereby disrupting the blood-brain barrier (BBB). Specific knockout or inhibition of TRPM2 expression in endothelial cells results in reduced infarct size, decreased immune cell infiltration, and suppressed oxidative stress. Thus, TRPM2 in endothelial cells may represent a safer and more effective therapeutic target for ischemic stroke (151). Additionally, the highly neurotoxic Zn^2+^ may also play a role in TRPM2-mediated I/R brain injury. Furthermore, the significantly neurotoxic Zn^2+^ may also contribute to TRPM2-mediated I/R brain injury. Ye et al. (152) demonstrated using both *in vivo* and ex vivo brain I/R models that elevated intracellular levels of ROS and Zn^2+^ following brain I/R are critical contributors to the death of pyramidal neurons in the hippocampal CA1 region. TRPM2 KO significantly inhibited the increase in intracellular Zn^2+^, reduced ROS levels, decreased the number of damaged or dead hippocampal pyramidal neurons, and markedly improved the 72-hour survival rate in mice, suggesting that TRPM2 activation leads to excessive intracellular Zn^2+^, which targets mitochondria, causing mitochondrial dysfunction and increased ROS production, ultimately resulting in neuronal death (152). Further studies have identified a positive correlation between infarct volume and bilirubin levels in both stroke patients and mouse stroke models. Bilirubin has been found to act as an endogenous agonist of TRPM2, specifically binding to and activating the channel, thereby exacerbating brain damage during ischemic stroke. Targeting the blockade of TRPM2 channels from binding with bilirubin may represent an effective therapeutic strategy to mitigate and prevent stroke-related brain injury (58).

Murat et al. (153) research found that renal I/R induces damage to both glomeruli and renal tubules in rats, leading to elevated levels of malondialdehyde, caspase-3 and oxidative stress index (OSI) within renal tissues. Concurrently, the activity of the antioxidant enzyme catalase (CAT) and the total antioxidant capacity decreased. Intraperitoneal administration of the TRPM2 inhibitor ACA resulted in a reduction of oxidative stress levels and an enhancement of antioxidant capacity in renal tissues, thereby improving the aforementioned renal I/R injuries. Research indicates that bilateral renal I/R induces tissue damage, functional impairment, inflammatory cell infiltration, and apoptosis in the kidneys of WT mice (154). However, in TRPM2-KO mice and those injected with 2-APB, there is an increased expression of anti-apoptotic proteins Bcl-2 and Bcl-xL, leading to significant mitigation of the aforementioned tissue damage. The study reveals a novel mechanism of TRPM2-mediated renal I/R injury, suggesting that I/R promotes an increase in Ras-related C3 botulinum toxin substrate 1(RAC1) levels and NOX activation in renal cells. RAC1 physically interacts with TRPM2, enhancing its expression, while also increasing NADPH oxidase activity. This cascade activates TRPM2 via the PARP/ADPR pathway, leading to apoptosis through the activation of caspase-3 and caspase-9, ultimately resulting in renal cell death and kidney injury (154).

Scholars have also investigated the role of TRPM2 in Hepatic I/R. TRPM2-mediated Ca^2+^ influx induces mitochondrial Ca^2+^ overload through the mitochondrial calcium uniporter (MCU), leading to an increase in the expression of arachidonate 12-lipoxygenase and the occurrence of mitochondrial lipid peroxidation and ferroptosis. Inhibition of TRPM2 by using a TRPM2 inhibitor alleviates such I/R-induced damage (155). Using TRPM2-deficient mice or siRNA-mediated TRPM2 knockdown mitigates hepatic I/R injuries (121, 156). These studies indicate that inhibiting the expression of TRPM2 may serve as an effective therapeutic strategy for diseases related to hepatic I/R injury, such as during liver transplantation.

### 6.2 The positive role of TRPM2 in cardiac I/R

However, other research suggests that the absence of TRPM2 exacerbates cardiac I/R injury. TRPM2 may protect the heart from I/R injury by improving mitochondrial dysfunction, maintaining cellular bioenergetics and reducing ROS levels (27, 28, 157, 158). Compared with healthy hearts, the expression of TRPM2 is significantly reduced in the cardiac tissues of patients with heart failure, indirectly supporting the hypothesis that TRPM2 has a cardioprotective effect (22). The expression of TRPM2 can also ameliorate doxorubicin-induced cardiac dysfunction and prolong the survival time of animals following doxorubicin intervention (158). Miller et al. (27)revealed that TRPM2 KO myocytes exhibited increased production of ROS when subjected to I/R. Simultaneously, antioxidant capacity significantly decreased, characterized by reduced expression of SOD and its upstream regulatory factors such as HIF-1α and FoxO3a; meanwhile, NADPH oxidase, which is involved in ROS generation, increased. These findings indicate that TRPM2 can protect myocytes from I/R and oxidative stress damage. This protective effect is associated with the reduction of ROS generation and enhanced antioxidant capacity in myocytes lacking TRPM2. Researchers employed proteomic techniques to explore the mechanisms by which the TRPM2 channel protects cardiomyocytes from I/R and oxidative stress damage. The most pronounced variances observed in canonical pathways between the hearts of mice with or without TRPM2 KO post I/R are associated with mitochondrial dysfunction and alterations in the tricarboxylic acid cycle. In TRPM2 KO mice, mitochondrial respiratory chain complexes I, III, and IV are downregulated, and the expression of complexes II and V is upregulated. The immunoblotting results confirmed the decreased expression of NDUFA4L2 and BNIP3. The myocardium of TRPM2 KO mice not only exhibited reduced ATP levels and oxygen consumption but also decreased ψm and a significant increase in ROS levels. The study suggests that the TRPM2 channel protects the heart from I/R injury by improving mitochondrial dysfunction and reducing ROS levels (157). Miller et al. (28) further investigated post I/R and WT mice hearts; the myocardium of TRPM2 KO mice exhibited a significant reduction in the expression of Pyk2 and its downstream pro-survival signaling molecules, pERK1/2, and pAkt. Following I/R in TRPM2 KO myocardial cells, ψm significantly decreased; patch clamp experiments revealed decreased activity of MCU, indicating a decline in mitochondrial Ca^2+^ uptake capacity. Simultaneously, ATP levels decreased, and ROS levels significantly increased. The activation of TRPM2-induced Ca^2+^ influx ameliorated these adverse reactions. Hence, TRPM2-mediated Ca^2+^ influx phosphorylates Pyk2, thereby enhancing mitochondrial calcium uptake and ATP generation capabilities after Pyk2 translocates to the mitochondria. Furthermore, Pyk2 protects the heart from I/R and oxidative stress injury by activating downstream pro-survival signaling pathways such as Akt and ERK1/2 (28).

**Figure 5:**
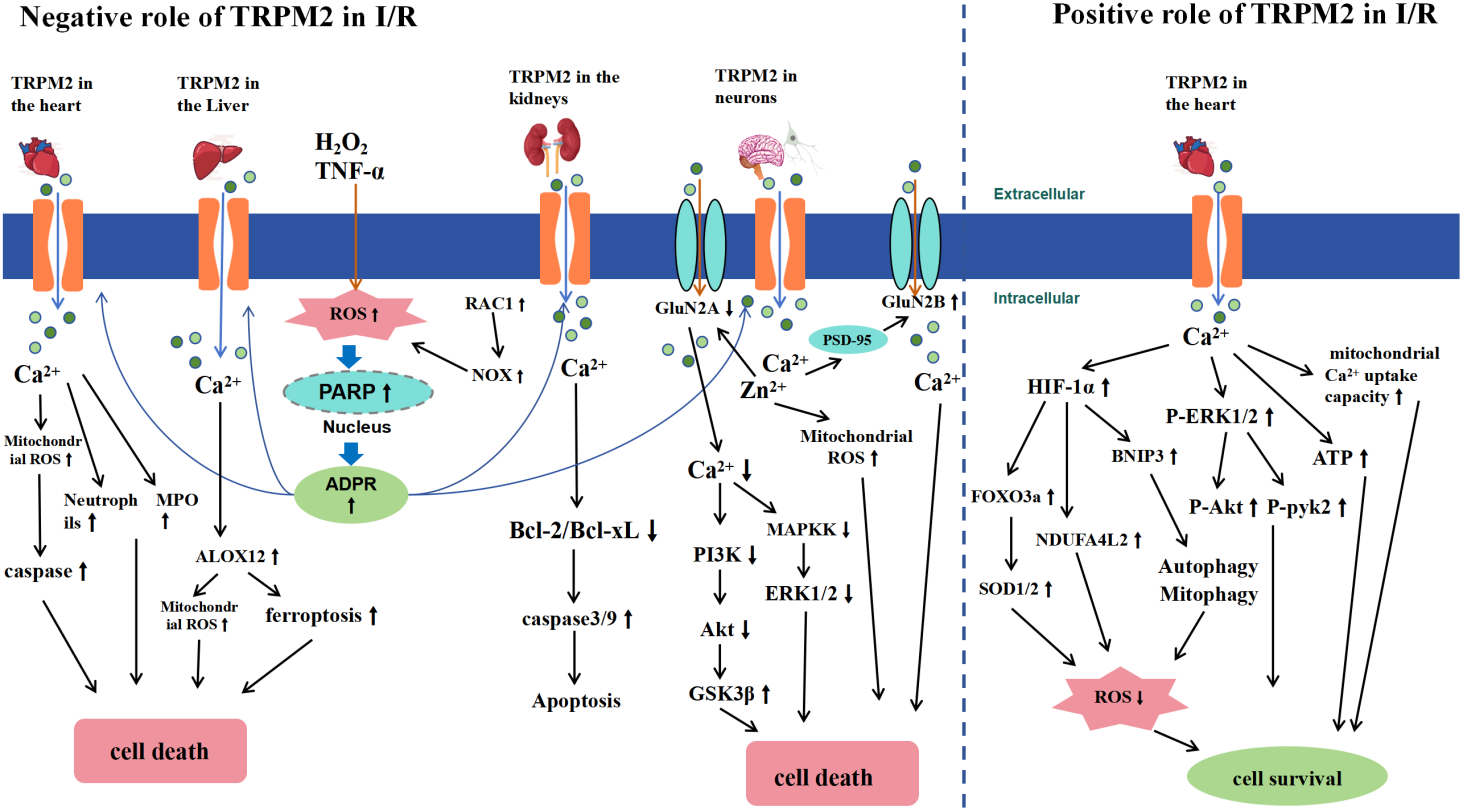
Schematic Representation of TRPM2-Mediated Signaling Pathways in Ischemia-Reperfusion (I/R) Injury. The left half of the diagram illustrates the negative effects of TRPM2 activation during I/R. I/R induces extracellular H2O2 or TNF-α, leading to intracellular ROS accumulation (RAC1-induced ROS accumulation occurs only in renal I/R). This accumulation activates nuclear PARP and PARG enzymes, converting NAD^+^ to ADPR, subsequently activating TRPM2. The activation of TRPM2 results in a significant influx of Ca^2+^ and Zn^2+^, which leads to cell death by increasing neutrophils, myeloperoxidase (MPO), and caspase in cardiomyocytes, inducing arachidonate 12-lipoxygenase (ALOX12) expression in the liver, increasing Bcl-2 and Bcl-xL expression in the kidney, and causing mitochondrial ROS accumulation and NMDA receptor activation in neurons, thereby inhibiting the downstream pro-survival signals of the Akt and ERK1/2 pathways. The right half of the diagram illustrates the positive effects of TRPM2 activation during I/R. TRPM2-mediated Ca^2+^ influx improves mitochondrial function, enhances ATP production, and reduces ROS levels by activating HIF-1α/-2α and ERK1/2 signaling pathways, thus protecting cardiomyocytes from I/R injury.

In summary, the TRPM2 channel plays a crucial role in I/R, that is, it exacerbates and mitigates effects on I/R injury (**Figure 5**). In brain I/R, as typified by ischemic stroke, TRPM2 activation has been found to mediate brain injury through multiple mechanisms. These include increasing neurotoxicity via direct and indirect interactions with NMDA receptors, disrupting the BBB through interactions with CD36, and causing intracellular Ca^2+^ and Zn^2+^ overload, leading to oxidative stress. In renal I/R, TRPM2 disrupts redox balance and activates the caspase cascade, resulting in apoptosis and renal cell death. In hepatic I/R, TRPM2 activation mediates Ca^2+^ overload, which is associated with lipid peroxidation and ferroptosis. Thus, inhibiting TRPM2 expression may be a potential therapeutic target for treating these I/R-related diseases, offering new directions and strategies for clinical treatment. In myocardial I/R, TRPM2 appears to have dual and opposing effects. Some researchers posit that TRPM2 is crucial for mediating I/R-induced myocardial injury. Conversely, other studies suggest that TRPM2 may protect tissues from I/R injury by regulating Ca^2+^ influx, improving mitochondrial function, maintaining bioenergetics, and reducing ROS levels. The role of TRPM2 in myocardial I/R remains a contentious issue. Current research suggests that discrepancies in experimental results may be attributed to several factors: differences in mouse I/R models, with longer I/R times potentially causing more severe myocardial damage; variations in assessment methods; and differing experimental conditions, such as the use of different anesthetics (pentobarbital and isoflurane) and surgical techniques (open vs. closed chest surgery in mice). Future research should aim to standardize experimental design and conditions to clarify the specific role of TRPM2 in myocardial I/R. Overall, TRPM2 channels exhibit diverse biological effects in I/R injury, making them a focal point in the development of therapeutic strategies for related diseases.

## 7 Conclusion and future perspectives

The role of TRPM2 in physiological and pathological processes such as oxidative stress, inflammation and I/R is complex and multifaceted. The activation of TRPM2 leads to cation influx and exerts a dual effect by modulating downstream pathways; that is, it exerts protective and deleterious effects during cellular injury. The observed differences, and even contradictory results, in the experiments are speculated to be associated with several factors. Firstly, cell types, animal models and experimental conditions varied across different studies. Secondly, the diverse functional roles and outcomes of the TRPM2 channel may exhibit tissue-specific differences. Additionally, the intricate biological networks and signaling pathways involved in processes such as oxidative stress, inflammation and I/R could contribute to the diverse effects of TRPM2 channel activation. Finally, discrepancies in research outcomes may be attributed to differences in research methodologies and experimental techniques, including distinct knockout methods and the use of various pharmacological agents for interventions. Achieving a comprehensive understanding of the physiological and pathological roles of TRPM2 requires consistent experimental designs and further in-depth investigations.

Future investigations into the bidirectional role of TRPM2 in oxidative stress, inflammation, and I/R are essential for understanding its specific mechanistic contributions to neurodegenerative diseases characterized by chronic oxidative stress and inflammation, inflammatory bowel diseases, and I/R-related conditions such as myocardial infarction and stroke. To better utilize TRPM2 channels for therapeutic interventions in relevant diseases, the development of specific TRPM2 channel inhibitors is imperative. For instance, the non-specific inhibitor 2-APB, commonly used in many experiments, also affects other TRP channels, either inhibiting (e.g., TRPV6) (159) or activating (e.g., TRPV2) (160) them, thus potentially compromising the accuracy and consistency of experimental outcomes. Recent studies have shown promising outcomes in experimental settings with newly synthesized TRPM2-specific inhibitors, as well as interfering peptides designed to disrupt TRPM2 interactions with specific proteins. These research findings are exciting as they provide powerful tools for further investigating the functional mechanisms of the TRPM2 pathway in future studies. Given the widespread expression of TRPM2 across various tissues and cells, including neurons, liver cells, cardiac myocytes, and immune cells, alongside the complex downstream signaling pathways associated with TRPM2, achieving a balance in the activation or inhibition of TRPM2 among different tissue cells to modulate physiological and pathological processes poses a challenging issue. Future research focusing on developing therapeutic strategies targeting specific intracellular signaling pathways in certain tissue cells holds great promise. Considering the bidirectional regulatory nature of TRPM2 channels, caution must be exercised in formulating treatment plans targeting TRPM2 as a therapeutic target until the precise effects of its activation or inhibition in different disease states are fully understood. In conclusion, research on TRPM2 channels is still in its exploratory phase, and future endeavors will further advance our understanding of this channel, providing new promising targets for the treatment of related diseases.

## Author contributions

PH: Writing – original draft, Writing – review & editing. CQ: Writing – review & editing. ZR: Writing – review & editing. DW: Writing – review & editing. JZ: Conceptualization, Funding acquisition, Supervision, Writing – review & editing.
